# Association between exposure to air pollutants and sleep parameters in chronic obstructive pulmonary disease patients with or without obstructive sleep apnea

**DOI:** 10.1097/CM9.0000000000002281

**Published:** 2022-08-24

**Authors:** Junyi Wang, Wanlu Sun, Wanzhou Wang, Wenlou Zhang, Ying Wang, Yongwei Huang, Jianli Wang, Liqiang Zhang, Yahong Chen, Xinbiao Guo, Furong Deng

**Affiliations:** 1 Department of Respiratory Medicine, Peking University Third Hospital, Beijing 100191, China; 2 Department of Respiratory and Critical Care Medicine, Beijing Institute of Respiratory Medicine and Beijing Chao-Yang Hospital, Capital Medical University, Beijing 100191, China; 3 Department of Occupational and Environmental Health Sciences, School of Public Health, Peking University, Beijing 100191, China.

*To the Editor*: Air pollution is a rapidly developing environmental problem worldwide. A large number of studies have indicated that ambient air pollutants have effects on mortality and morbidity of respiratory diseases. Remarkably, paramount attention should be paid to the impact on respiratory diseases associated with air pollution from a public health standpoint. Chronic obstructive pulmonary disease (COPD) is a common chronic respiratory disease characterized by partially irreversible chronic airflow limitation that may lead to disability and impaired quality of life.^[1,2]^ The most recent national survey in China reports that the overall prevalence of COPD patients aged ≥40 years has reached 13.7% in 2012 to 2015.^[3]^ Obstructive sleep apnea (OSA), one of the common complications of COPD, is characterized by repetitive episodes of partial or complete upper airway obstruction during sleep, leading to sleep apnea-hypopnea, hypoxemia, and sleep fragmentation.

Given that the detrimental effects of air pollutants on COPD have received increasing attention recently, it is particularly important to explore whether there is a link between air pollutant exposure and the likelihood of coexisting OSA among COPD patients.

This cross-sectional, observational study was based on COPD outpatients enrolled in Peking University Third Hospital during November 2014 and May 2018 in Beijing, China. The study was conducted with the approval of the Ethics Committee of Peking University Third Hospital (No. IRB00006761-M2016164), and the guidelines outlined in the *Declaration of Helsinki* were followed. All patients signed informed consent before the investigation. The COPD was defined by reviewing the medical records for a clinical diagnosis of COPD and spirometric data meeting the diagnostic criteria of the Global Initiative for Chronic Obstructive Lung Disease (GOLD) guidelines (2017).^[4]^ The spirometric criterion for COPD is a postbronchodilator fixed ratio of forced expiratory volume in 1 s (FEV_1_)/forced vital capacity (FVC) <0.70. Patients were screened and enrolled based on the following selection criteria: (1) age ≥40 years; (2) stable COPD outpatients with no acute exacerbation of symptoms and upper respiratory tract infection in the 6 weeks preceding the study; (3) living in Beijing for ≥1 year. We excluded (1) patients with neurological or psychiatric disorders, who were thus unable to complete the questionnaire; (2) patients with asthma, interstitial lung disease, pulmonary tuberculosis, lung cancer, or any other respiratory disease; (3) patients who were taking drugs that may affect sleep; and (4) patients with central sleep apnea syndrome, which may interfere with the diagnosis of OSA.

Daily average concentrations of six major air pollutants during the study period were collected from the China National Environmental Monitoring Center (http://www.cnemc.cn/), including the 24-h average concentration of particulate matter with aerodynamic diameter <2.5 μm (PM_2.5_), particulate matter with aerodynamic diameter <10 μm (PM_10_), sulfur dioxide (SO_2_), carbon monoxide (CO), nitrogen dioxide (NO_2_), and daily maximum 8-h-ozone (O_3_).

ApneaLink^TM^ (ResMed, MAP Medicine Technology,Martinsried, Germany) monitor was used for recording the sleep parameters, including the nasal respiratory pressure signal, thoracoabdominal movement, and oxygen saturation (SpO_2_). All physiologically important sleep parameters were identified according to the 2017 American Academy of Sleep Medicine.^[5]^ Participants were allocated into either the COPD with OSA group or into the COPD without OSA group based on whether home portable monitoring evaluation revealed an apnea-hypopnea index (AHI) of ≥5 events/h or <5 events/h, respectively.

Continuous variables with normal distribution were presented as mean ± standard deviation and those with skewed distribution were expressed as median (interquartile range [IQR]). Categorical variables were shown as numbers and percentages. Chi-squared test and Student's *t* test or Mann-Whitney *U* test were used to examine the subgroup differences. The multiple linear regression (MLR) model was used to evaluate the effects of ambient air pollution on sleep parameters among COPD patients with or without OSA, adjusting for gender, age, body mass index (BMI), smoking status (never smoker, former smoker, and current smoker), occupational exposure (yes or no), cooking oil fumes exposure (yes or no), GOLD stage (GOLD 1, 2, 3, or 4), temperature, and relative humidity (RH). Cumulative lags (from lag 01 to lag 07) were utilized to assess the effect estimates for each air pollutant. The results were reported as percentage changes in sleep parameters and 95% confidence intervals (CIs) per IQR in air pollutant concentrations. R 3.6.1 (https://www.r-project.org/) was used for statistical analysis. Statistical significance was defined as two-sided *P* < 0.05.

This study investigated 109 COPD cases. There were 45(41.3%) subjects (36 males and 9 females) in the COPD without OSA group and 64 (58.7%) subjects (58 males and 6 females) in the COPD with OSA group. The demographic and clinical characteristics of the subjects were presented in Supplementary Table 1. AHI was significantly higher in COPD-with-OSA patients compared to COPD-without-OSA patients (19.5 [22.8] events/h *vs*. 5.0 [4.5] events/h, *P* < 0.001). Min-SpO_2_ and mean-SpO_2_ were significantly lower in COPD with OSA patients *vs*. COPD without OSA patients [Supplementary Figure 1].

The average concentrations of air pollutants and meteorological data were presented in Supplementary Table 2. Spearman's correlation analysis showed positive correlations between particulate matter, gaseous pollutants (SO_2_, NO_2_, and CO) and RH, with coefficients ranging from 0.256 to 0.876 [Supplementary Table 3].

The MLR model showed significant associations were found between exposure to PM_2.5_, PM_10_, O_3_, SO_2_, and oxygen desaturation index (ODI) [Supplementary Figure 2]. It is observed that IQR increases in PM_2.5_ at lag05, PM_10_ at lag07, O_3_ at lag07, and SO_2_ at lag07 were associated with 0.83% (95% CI: 0.30%, 1.36%), 0.53% (95% CI: 0.04%, 1.03%), 1.84% (95% CI: 0.60%, 3.06%), and 1.68% (95% CI: 0.62%, 2.73%) increases in ODI, respectively. Significantly negative associations were also found between air pollutants (PM_2.5_ and PM_10_) and SpO_2_ [Supplementary Figure 3]. Per IQR increase in PM_2.5_ at lag01 was associated with a −0.020% (95% CI: −0.035%, −0.002%) change in base SpO_2_. An IQR increase in PM_10_ at lag06 were related with a −0.014% (95% CI: −0.028%, −0.001%) change in base-SpO_2_ and a −0.570% (95% CI: −1.090%, −0.048%) change in min-SpO_2_, respectively. Additionally, IQR increase in PM_2.5_ and PM_10_ at lag04 were associated with a 0.90% (95% CI: 0.22%, 1.58%) and 0.66% (95% CI: 0.10%, 1.22%) increase in percentage of total sleep time with saturation <90% (T90), respectively [Supplementary Figure 4].

The MLR model also found more significant associations between exposure to air pollutants (PM_2.5_, PM_10_, O_3_, NO_2_, and SO_2_) and sleep parameters (such as ODI, SpO_2_, and T90) in the COPD with OSA patients than those in COPD without OSA patients [Figure 1]. In COPD patients with OSA, the results found that an IQR increase in PM_10_ at lag03 was associated with a 0.67% (95% CI: 0.13%, 1.59%) increase in ODI and a −0.74% (95% CI: −1.40%, −0.08%) change in min SpO_2_, respectively. IQR increases in O_3_ at lag0, NO_2_ at lag0, and SO_2_ at lag0 were associated with 2.99% (95% CI: 0.32%, 5.95%), 3.38% (95% CI: 0.64%, 6.12%), and 3.44% (95% CI: 1.09%, 6.20%) increases in T90, respectively. Per IQR increase in SO_2_ at lag0 was associated with a −2.82% (95% CI: −5.88%, −0.22%) change in base SpO_2_. Sensitivity analysis showed consistency with the overall analysis [Supplementary Table 4].

**Figure 1:**
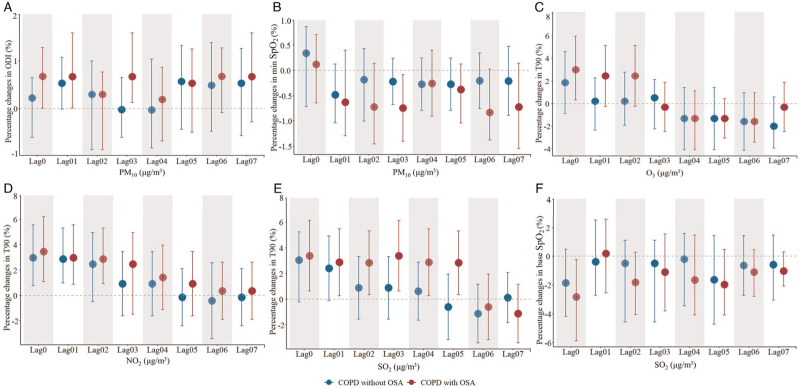
Estimated percentage changes of sleep parameters with an IQR increase in air pollutants among COPD patients with or without OSA. (A) PM_10_–ODI; (B) PM_10_–min SpO_2_; (C) O_3_–T90; (D) NO_2_–T90; (E) SO_2_–T90; and (F) SO_2_–base SpO_2_. The MLR model adjusted for confounders including gender, age, body mass index (BMI), smoking status, occupational exposure, cooking oil fumes exposure, GOLD stage, temperature, and RH. COPD: Chronic Obstructive Pulmonary Disease; GOLD: Global initiative for chronic obstructive lung disease; IQR: Interquartile range; MLR: Multiple linear regression; NO_2_: Nitrogen dioxide; O_3_: Ozone; ODI: Oxygen desaturation index; OSA: Obstructive sleep apnea; PM_10_: Particulate matter with aerodynamic diameter 10 μm; RH: Relative humidity; SO_2_: Sulfur dioxide; SpO_2_: Oxygen saturation; T90: Percentage of total sleep time with oxygen saturation <90%.

This cross-sectional study sought to explore the associations between exposure to air pollutants (PM_2.5_, PM_10_, O_3_, and SO_2_) and sleep parameters among COPD patients with or without OSA. Increased air pollutant concentrations were associated with elevated ODI and T90, as well as declined SpO_2_. ODI and T90 reflect nocturnal hypoxemia, which is related to OSA severity. Further, the stronger effects might be more robust in COPD with OSA patients. Our results found a more significant association between exposure to air pollutants and reduced SpO_2_ among COPD patients with OSA. COPD with OSA patients suffered from more frequent sleep-related symptoms due to abnormalities of upper airway collapse than COPD without OSA patients during nocturnal sleep. This might be attributable to the hypothesis that air-pollutant-mediated declines in SpO_2_ could cause sleep-related symptoms and ultimately lead to adverse effects in the long run, which means SpO_2_ appears to be an essential risk factor for adverse health outcomes among COPD patients.

Generally, air pollutants are thought to affect sleep outcomes via central nervous system regulation and/or via changes in respiratory system physiology.^[6]^ The reason may be that the deposition of pollutant particles in the airways directly contributes to nasal or pharyngeal inflammatory swelling of local mucous membranes, leading to reduced airway patency and thus increase in upper airway resistance. Meanwhile, another assumption is that the biochemistry of the central nervous system may be directly affected by air pollutants via the olfactory nerve, resulting in altered expression and dysregulation of neurochemicals. Specifically, air pollutants into the brain have been hypothesized to alter serotonin levels, induce the breakdown of protective epithelial barriers, and damage nerve cells.

In 2013 to 2017, China launched the nationwide Air Pollution Prevention and Control Action Plan to overcome the air quality problems. Although remarkable improvements in air quality have been made during this study period, the air pollutants concentrations were still higher than World Health Organization guidelines, particularly for particulate matter. Therefore, this study adds concrete evidence that COPD patients can potentially benefit from improved air quality in developing countries facing the problem of relatively severe air pollution. Further, for those COPD patients with OSA who face regular intermittent high-level air pollution exposure, we recommend personal protective measures to help reduce respiratory health effects.

In conclusion, COPD with OSA patients demonstrated more significant changes in sleep parameters with exposure to air pollutants than those in COPD without OSA patients. The results indicate that air quality-targeted environmental strategies should be implemented to mitigate the effects of air pollution on COPD patients.

## Funding

This work was supported by the Capital Health Development Research Project (No. 2020-2Z-40917) and the National Natural Science Foundation of China (Nos. 82090014, 22076006, and 91543112).

## Conflicts of interest

None.

## Supplementary Material

Supplemental Digital Content
