# Supplementary material for: Association between exposure to air pollutants and sleep parameters in chronic obstructive pulmonary disease patients with or without obstructive sleep apnea
Source: Chin Med J (Engl). 2022 Aug 24;135(16):2014–6. doi: 10.1097/CM9.0000000000002281 (PMC9746724; doi:10.1097/CM9.0000000000002281)
Supplement: Supplemental Digital Content [file cm9-135-2014-s001.docx]

**Supplementary Table 1: Basic characteristics of COPD patients with or without OSA.**

| **Characteristics** | **Total** | **COPD without OSA** | **COPD with OSA** | **Statistics** | ***P*-value*^*^*** |
| --- | --- | --- | --- | --- | --- |
| Number of participants, *n* (%) | 109 (100.0) | 45 (41.3) | 64 (58.7) |  |  |
| Gender, *n* (%) |  |  |  | 2.513^*^ | 0.158 |
| Male | 94 (86.2) | 36 (38.3) | 58 (61.7) |  |  |
| Female | 15 (13.8) | 9 (60.0) | 6 (40.0) |  |  |
| Age (years), Median (IQR) | 66.0 (12.5) | 68.0 (11.0) | 65.0 (13.3) | 0.240^†^ | 0.549 |
| BMI (kg/m^2^), Median (IQR) | 23.9 (4.5) | 23.5 (5.1) | 24.0 (4.5) | 0.398^†^ | 0.562 |
| Smoking status, *n* (%) |  |  |  | 5.105^*^ | 0.078 |
| Never smoker | 14 (12.8) | 4 (28.6) | 10 (71.4) |  |  |
| Former smoker | 68 (62.4) | 25 (36.8) | 43 (63.2) |  |  |
| Current smoker | 27 (24.8) | 16 (59.3) | 11 (40.7) |  |  |
| Smoke packs per year (packs/year), Median (IQR) | 31.7 (34.2) | 25.0 (50.0) | 21.5 (38.5) | 0.670^†^ | 0.475 |
| Cooking oil fumes, *n* (%) |  |  |  | 0.417^*^ | 0.539 |
| No | 74 (67.9) | 29 (39.2) | 45 (60.8) |  |  |
| Yes | 35 (32.1) | 16 (45.7) | 19 (52.7) |  |  |
| Occupation exposure, *n* (%) |  |  |  | 1.999^*^ | 0.172 |
| No | 64 (58.7) | 30 (46.9) | 34 (53.1) |  |  |
| Yes | 45 (41.3) | 15 (33.3) | 30 (66.7) |  |  |
| Drug therapy, *n* (%) |  |  |  | 12.918^*^ | 0.012 |
| LAMA | 15 (13.8) | 7 (46.7) | 8 (53.3) |  |  |
| LABA + ICS | 33 (30.3) | 11 (33.3) | 22 (66.7) |  |  |
| LAMA + LABA + ICS | 41 (37.6) | 12 (29.3) | 29 (70.7) |  |  |
| Theophylline | 13 (11.9) | 10 (76.9) | 3 (23.1) |  |  |
| Other drugs | 7 (6.4) | 5 (71.4) | 2 (28.6) |  |  |
| Lung function, Median (IQR) |  |  |  |  |  |
| FVC (L) | 2.9 (1.0) | 3.3 (1.2) | 2.8 (1.0) | 0.722^†^ | 0.102 |
| FEV_1_ (L) | 1.9 (1.1) | 2.1 (1.2) | 1.9 (1.1) | 0.367^†^ | 0.998 |
| FEV_1_/FVC (%) | 77.9 (25.2) | 82.0 (19.5) | 76.1 (29.2) | 0.051^†^ | 0.828 |
| GOLD stage, *n* (%) |  |  |  | 1.334^*^ | 0.721 |
| GOLD 1 | 24 (22.0) | 9 (37.5) | 15 (62.5) |  |  |
| GOLD 2 | 54 (49.5) | 25 (46.3) | 29 (53.7) |  |  |
| GOLD 3 | 24 (22.0) | 8 (33.3) | 16 (66.7) |  |  |
| GOLD 4 | 7 (6.4) | 3 (42.9) | 4 (57.1) |  |  |

^*^*χ*^2^ value; ^†^*Z* value. BMI: Body mass index; COPD: Chronic obstructive pulmonary disease; FEV_1_: Forced expiratory volume in 1 s; FVC: Forced vital capacity; GOLD: Global Initiative for Chronic Obstructive Lung Disease; ICS: Inhaled corticosteroids; LABA: Long-acting beta 2 agonists; LAMA: Long-acting antimuscarinic antagonists; IQR: Interquartile range; OSA: Obstructive sleep apnea.

**Supplementary Table 2: Distributions of daily average concentrations of air pollutants and meteorological data.**

| **Air pollutants** | **Mean ± SD** | **Min** | **P_25_** | **P_50_** | **P_75_** | **Max** | **IQR** |
| --- | --- | --- | --- | --- | --- | --- | --- |
| PM_2.5_ (μg/m^3^) | 64.6 ± 67.5 | 5.0 | 23.5 | 39.0 | 83.5 | 364.0 | 60.0 |
| PM_10_ (μg/m^3^) | 93.3 ± 80.6 | 0 | 43.5 | 72.0 | 118.5 | 550.0 | 75.0 |
| SO_2_ (μg/m^3^) | 9.2 ± 8.9 | 2.0 | 3.0 | 6.0 | 11.0 | 42.0 | 8.0 |
| CO (mg/m^3^) | 1.1 ± 1.1 | 0.2 | 0.5 | 0.8 | 1.1 | 7.5 | 0.6 |
| NO_2_ (μg/m^3^) | 48.5 ± 26.5 | 10.0 | 31.0 | 41.0 | 53.0 | 153.0 | 22.0 |
| O_3_ (μg/m^3^) | 92.3 ± 65.7 | 0 | 43.5 | 74.0 | 141.5 | 262.0 | 98.0 |
| Temperature (°C) | 11.8 ± 11.4 | -4.0 | 1.0 | 11.0 | 23.5 | 31.0 | 22.5 |
| RH (%) | 48.0 ± 18.5 | 14.0 | 35.0 | 44.0 | 64.5 | 88.0 | 29.5 |

CO: Carbon monoxide; IQR: Interquartile range; Max: Maximum; Min: Minimum; NO_2_: Nitrogen dioxide; O_3_: Ozone; PM_10_: Particulate matter with aerodynamic diameter <10 μm; PM_2.5_: Particulate matter with aerodynamic diameter <2.5 µm; RH: Relative humidity; SD: Standard deviation; SO_2_: Sulfur dioxide.

**Supplementary Table 3: Spearman’s correlation coefficients among air pollutants and meteorological parameters.**

| **Air pollutants** | PM_2.5_ | PM_10_ | SO_2_ | CO | NO_2_ | O_3_ | Temperature | RH |
| --- | --- | --- | --- | --- | --- | --- | --- | --- |
| PM_2.5_ | 1.000 | 0.837^‡^ | 0.625^‡^ | 0.876^‡^ | 0.682^‡^ | -0.105 | -0.055 | 0.568^‡^ |
| PM_10_ |  | 1.000 | 0.625^‡^ | 0.712^‡^ | 0.699^‡^ | 0.012 | -0.014 | 0.306^‡^ |
| SO_2_ |  |  | 1.000 | 0.688^‡^ | 0.689^‡^ | -0.389^‡^ | -0.508^‡^ | -0.036 |
| CO |  |  |  | 1.000 | 0.717^‡^ | -0.282^†^ | -0.208^*^ | 0.544^‡^ |
| NO_2_ |  |  |  |  | 1.000 | -0.401^‡^ | -0.352^‡^ | 0.265^†^ |
| O_3_ |  |  |  |  |  | 1.000 | 0.854^‡^ | -0.014 |
| Temperature |  |  |  |  |  |  | 1.000 | 0.256^†^ |
| RH |  |  |  |  |  |  |  | 1.000 |

CO: Carbon monoxide; NO_2_: Nitrogen dioxide; O_3_: Ozone; PM_10_: Particulate matter with aerodynamic diameter <10 μm; PM_2.5_: Particulate matter with aerodynamic diameter <2.5 µm; RH: Relative humidity; SO_2_: Sulfur dioxide. ^*^*P*<0.05; ^†^*P*<0.01; ^‡^*P*<0.001.

**Supplementary Table 4: Associations between ambient air pollutants and sleep parameters in two-pollutant models.**

| Sleep parameters | Air pollutants | Two-pollutant model with PM_2.5_ | Two-pollutant model with PM_10_ | Two-pollutant model with NO_2_ | Two-pollutant model with SO_2_ | Two-pollutant model with O_3_ | Two-pollutant model with CO |
| --- | --- | --- | --- | --- | --- | --- | --- |
| ODI | PM_2.5_-lag05 |  | 0.87% (-0.02%,1.77%) | 0.74% (-0.22%,1.71%) | 0.68% (-0.22%,1.59%) | 0.65% (-0.22%,1.52%) | 0.26% (-0.76%,1.28%) |
|  | PM_10_-lag07 | 0.62% (-0.57%,1.81%) |  | 0.12% (-0.17%,0.40%) | 0.1% (-0.18%,0.39%) | -0.52% (-1.02%,0.02%) | 0.06% (-0.23%,0.35%) |
|  | SO_2_-lag07 | 6.12% (-8.43%,22.99%) | 9.02% (-5.56%,25.85%) | 8.4% (-6.14%,25.20%) |  | 8.02% (-6.59%,24.92%) | 3.89% (-10.62%,20.74%) |
|  | O_3_-lag07 | -0.26% (-1.07%, 0.54%) | -0.29% (-1.11%,0.52%) | -0.59% (-1.46%,0.23%) | 0.28% (-0.53%,1.10%) |  | -0.28% (-1.09%,0.53%)^*^ |
|  |  |  |  |  |  |  |  |
| Base SpO_2_ | PM_2.5_-lag01 |  | -0.01% (-0.04%,0.04%) | 0.002% (-0.16%,0.02%) | 0.01% (-0.004%,0.02%) | 0.01% (-0.04%,0.019%) | -0.012% (-0.04%,0.014%) |
|  | PM_10_-lag06 | 0.18% (-0.15%,0.50%) |  | 0.23% (-0.05%,0.52%) | 0.22% (-0.06%,0.51%) | 0.21% (-0.07%,0.50%) | 0.19% (-0.10%,0.47%) |
|  |  |  |  |  |  |  |  |
| Min SpO_2_ | PM_10_-lag06 | -0.88% (-2.49%,0.72%) |  | 0.14% (-0.15%,0.43%) | 0.13% (-0.16%,0.42%) | 0.13% (-0.16%,0.27%) | 0.09% (-0.20%,0.38%) |
|  |  |  |  |  |  |  |  |
| T90 | PM_2.5_-lag04 |  | -1.13% (-3.03%,0.77%) | -1.15% (-2.43%, -0.12%) | -0.93% (-1.85%, -0.02%) | -0.92% (-1.62%, -0.21%) | -0.13% (-1.88%, 1.62%) |
|  | PM_10_-lag04 | 0.20% (-1.35%,1.76%) |  | -0.63% (-1.62%, 0.34%) | -0.58% (-1.26%,0.09%) | -0.66% (-1.24%, -0.09%) | 0.26% (-0.76%,1.28%) |

CIs: Confidence intervals; CO: Carbon monoxide; NO_2_: Nitrogen dioxide; O_3_: Ozone; ODI: Oxygen desaturation index; PM_10_: Particulate matter with aerodynamic diameter <10 μm; PM_2.5_: Particulate matter with aerodynamic diameter <2.5 µm; SO_2_: Sulfur dioxide; SpO_2_: Oxygen saturation; T90: Percentage of total sleep time with oxygen saturation < 90%.


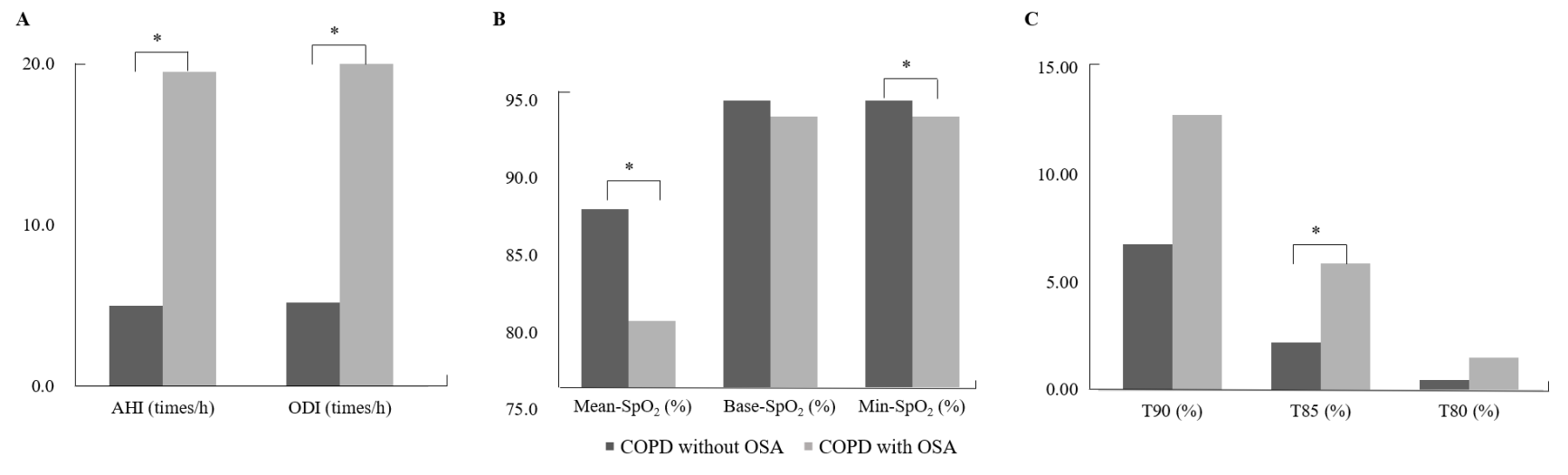


**Supplementary Figure 1:** The differences in health indicators AHI and ODI (A), SpO_2_ (B) and T90, T85 and T80 (C) of COPD with or without OSA group. AHI: Apnea-hypopnea Index; COPD: Chronic Obstructive Pulmonary Disease; ODI: Oxygen Desaturation Index; OSA: Obstructive sleep apnea; SpO_2_: Oxygen saturation; T80: Percentage sleep time with < 80% saturation; T85: Percentage sleep time with < 85% saturation; T90: Percentage of total sleep time with oxygen saturation < 90%. ^*^*P* <0.05.

**
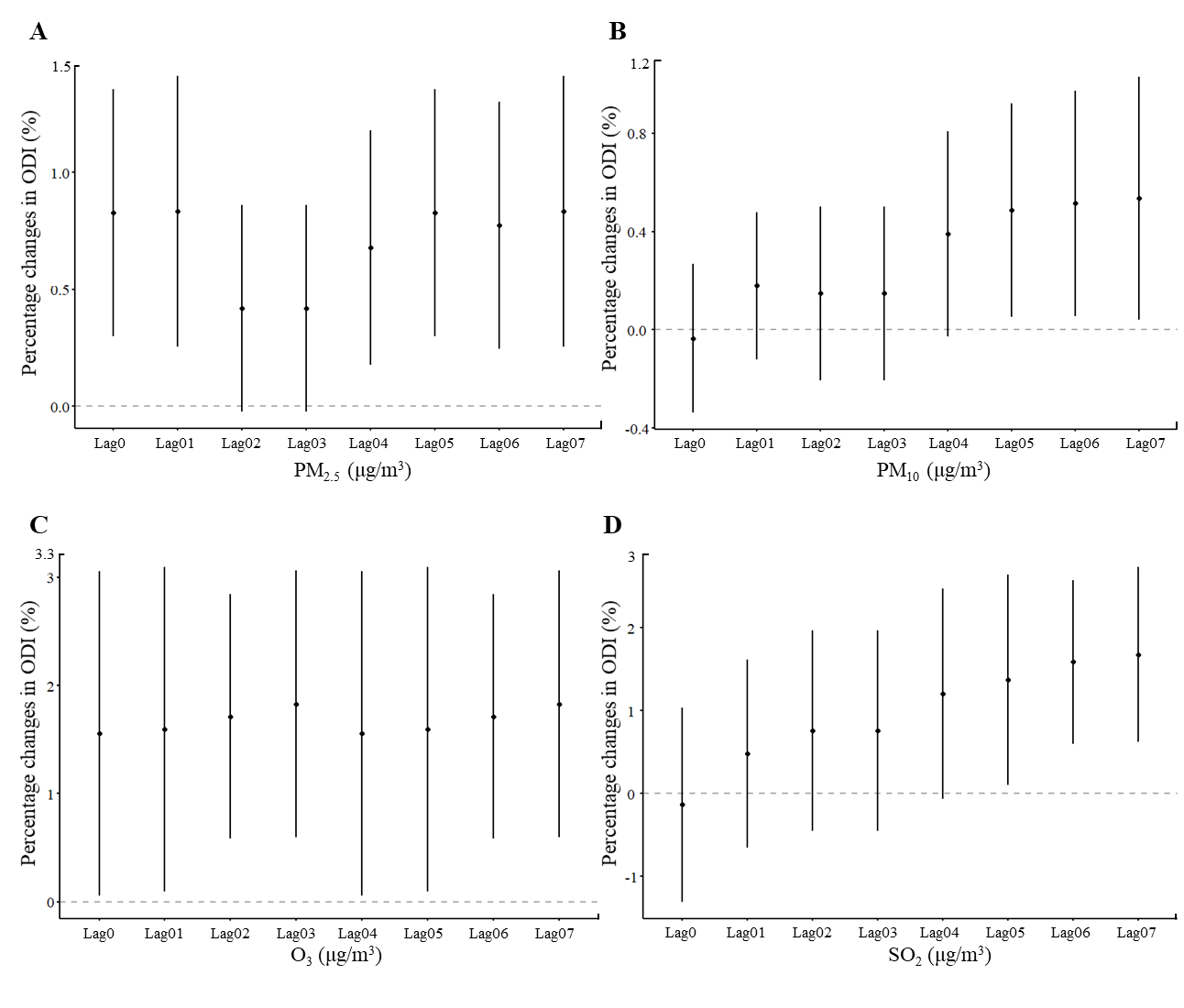
**

**Supplementary Figure 2:** Associations between exposure to air pollutants and ODI among all participants using MLR model (an IQR increase in PM_2.5_ [A], PM_10_ [B], O_3_ [C] and SO_2_ [D]). The MLR model adjusted for confounders including gender, age, body mass index (BMI), smoking status, occupational exposure, cooking oil fumes exposure, GOLD stage, temperature, and RH. GOLD: Global initiative for chronic obstructive lung disease; IQR: Interquartile range; MLR: Multiple linear regression; O_3_: Ozone; ODI: Oxygen desaturation index; PM_10_: Particulate matter with aerodynamic diameter <10 μm; PM_2.5_: Particulate matter with aerodynamic diameter <2.5 µm; RH: Relative humidity; SO_2_: Sulfur dioxide.


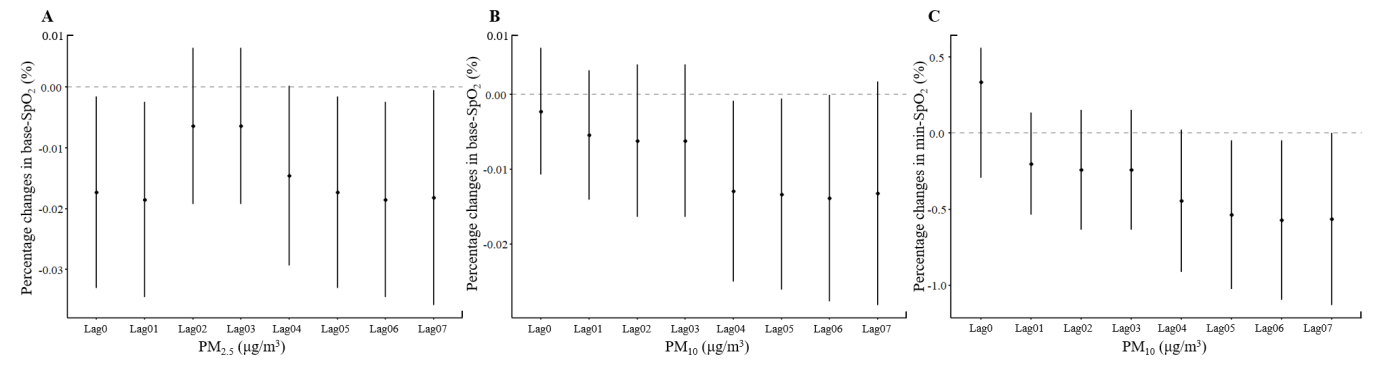


**Supplementary Figure 3:** Associations between exposure to PM_2.5_ and base-SpO_2_ (A), PM_10_ and base-SpO_2_ (B), and PM_10_ and min-SpO_2_ (C) among all participants using MLR model (an IQR increase in PM_2.5_, PM_10_). The MLR model adjusted for confounders including gender, age, body mass index (BMI), smoking status, occupational exposure, cooking oil fumes exposure, GOLD stage, temperature, and RH. GOLD: Global initiative for chronic obstructive lung disease; IQR: Interquartile range; MLR: Multiple linear regression; PM_10_: Particulate matter with aerodynamic diameter <10 μm; PM_2.5_: Particulate matter with aerodynamic diameter <2.5 µm; RH: Relative humidity; SpO_2_: Oxygen saturation.


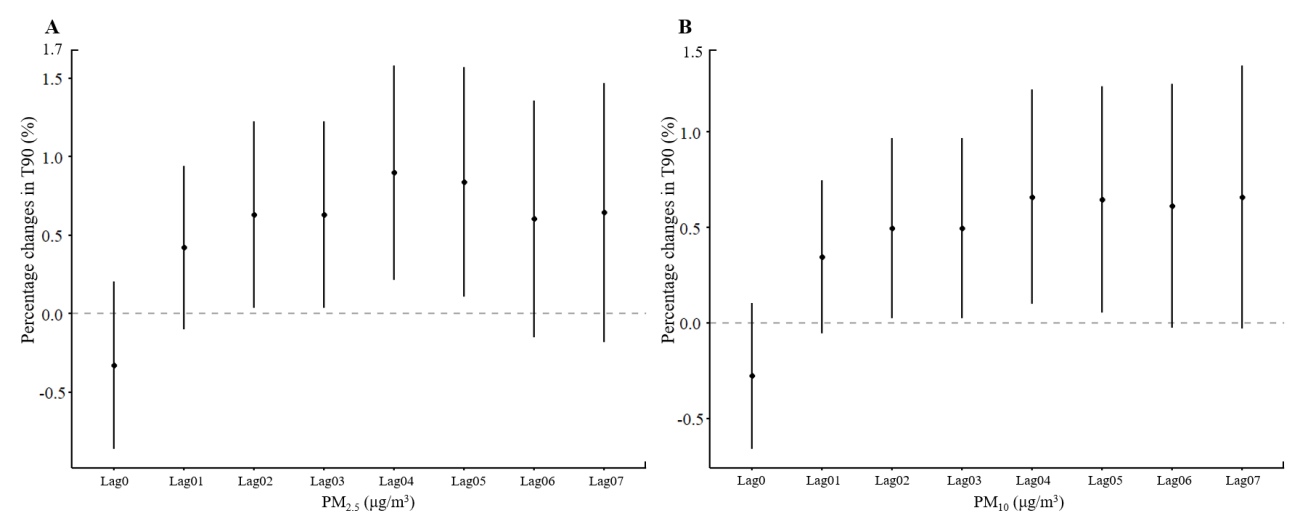


**Supplementary Figure 4:** Associations between exposure to air pollutants PM_2.5_ (A), PM_10_ (B) and T90 among all participants using MLR model (an IQR increase in PM_2.5_, PM_10_). The MLR model adjusted for confounders including gender, age, body mass index (BMI), smoking status, occupational exposure, cooking oil fumes exposure, GOLD stage, temperature, and RH. GOLD: Global initiative for chronic obstructive lung disease; IQR: Interquartile range; MLR: Multiple linear regression; PM_10_: Particulate matter with aerodynamic diameter <10 μm; PM_2.5_: Particulate matter with aerodynamic diameter <2.5 µm; RH: Relative humidity; T90: Percentage of total sleep time with oxygen saturation < 90%.
